# Cost of a lymphedema treatment mandate - 16 years of experience in the Commonwealth of Virginia

**DOI:** 10.1186/s13561-022-00388-6

**Published:** 2022-07-23

**Authors:** Robert Weiss

**Affiliations:** Independent Lymphedema Patient Advocate, 10671 Baton Rouge Avenue, Porter Ranch, CA 91326 USA

**Keywords:** Lymphedema treatment costs, Health care costs, Health insurance, Medical claims, Insurance mandates, Chronic disease management, Economic analysis, Treatment benefits

## Abstract

**Background:**

Treatment of chronic illness accounts for over 90% of Medicare spending. Chronic lymphedema places 3–10 million Americans at risk for recurrent cellulitis. Without convincing predictions of the costs and benefits of lymphedema treatment, insurers are reluctant to fully cover treatment of this common condition. Earlier papers discussed the costs and benefits of the first 5, 7, and 10 years of a lymphedema treatment mandate in Virginia. This paper updates these costs and benefits to 16 years of experience, and includes the impacts of the Patient Protection and Affordable Care Act of 2010 and the transition to ICD-10-CM diagnostic codes in 2015. It provides added confidence that costs of a lymphedema treatment mandate are reasonable, and can result in health insurance contract savings for reduced medical visits and hospitalizations for lymphedema patients.

**Methods:**

Virginia requires annual reporting of the segregated costs of each of its 30 medical mandates. Data on Virginia’s lymphedema treatment mandate for the years 2004 to 2019 have been collected from the series of annual reports. These data include actual lymphedema treatment claims data, utilization data, and claims-based estimates of the premium impact.

**Results:**

The average actual lymphedema claim cost was $2.03 per individual contract and $3.54 per group contract for the years reported, representing 0.05 and 0.08% of average total claims. The estimated premium impact was 0.16–0.32% of total average premium for all mandated coverage contracts. While lymphedema claim costs increased 3–6% per year over the study period, generally following the rise of health care costs, claim costs as a percent of average contract claims fell at a rate of 1.26–1.52% per year over that period. Medical office visits for lymphedema-related services fell from 0.10 to 0.02 visits per year per contract from the beginning to the end of the study period, and hospitalizations for lymphedema or lymphedema-related cellulitis fell to almost zero.

**Conclusions:**

The Virginia data confirmed previous conclusions that the costs of treatment of lymphedema are a small part of a typical health insurance contract, and that treatment of lymphedema by managing swelling results in lower overall medical costs and fewer hospitalizations. This is a potent model for reduction in healthcare costs while improving the quality of care for cancer survivors and others suffering with this chronic progressive condition.

## Background

One of our most urgent societal problems today is our inability to afford quality healthcare [1]. In a 2015 letter to Medicare patients with chronic conditions U.S. Senator Orrin G. Hatch, Chairman of the Senate Committee on Finance, stated “Treatment of chronic illnesses now accounts for almost 93 percent of Medicare spending”. Lymphedema, once acquired, is a lifelong disease with no currently known cure. If left untreated it is progressive, advancing in severity to less-easily treatable stages [2].

Cellulitis is a common global health burden, with more than 650,000 admissions per year in the United States, where an estimated 14.5 million cases annually of cellulitis account for $3.7 billion in ambulatory care costs alone [3]. The goals of lymphedema treatment in all stages are to prevent progression of lymphedema and to avoid lymphedema-related infections (e.g. cellulitis, lymphangitis, erysipelas, dermatolymphangioadenitis). Lymphedema is a risk factor for cellulitis, and tissue damage due to cellulitis is a risk factor for lymphedema [4–6]. This strong relationship between lymphedema and cellulitis forms the basis for the proposition that management of lymphedema can provide a mechanism for reducing the significant medical costs of cellulitis hospitalizations.

Decades of literature have described how treatment of lymphedema by reduction in swelling is effective in reducing progression of lymphedema, reducing infection and hospitalizations, and preventing functional disability [7–10]. However, the quality of available treatment often does not meet the recommended standards of knowledgeable lymphedema specialty groups such as the International Society of Lymphology (ISL) [11]. Non-surgical conservative treatment standards include an intensive clinical treatment phase by specially-qualified therapists, including, as required: manual lymph drainage; multilayered bandages; range-of-motion exercises; and patient instruction in self-treatment. The home care maintenance phase may include: the provision and daily use of low-stretch compression garments and devices; meticulous skin care; decongestion exercises; and repeated light massage as required [11]. Insurance coverage of these elements of treatment is sporadic, often driven by “pound-foolish” fiscal policies that ignore the preventive value of early intervention and effective home management.

A protocol was proposed for a prospective surveillance model comprising screening for preclinical lymphedema followed by appropriate intervention with the aim of preventing development into clinical lymphedema and cellulitis [12]. A later prospective trial confirmed that this approach could result in significant cost savings [13].

Clinical capability for the screening and diagnosis of preclinical lymphedema began to appear in the early 2000’s [14] and continues to date [15, 16]. Some private insurers reimburse for lymphedema screening assessments. Modelling studies using claims data over the period 2012–2016 further suggested that extension of these available early diagnosis and edema-treatment methods to treat lymphedema patients with comorbid chronic venous insufficiency and recurrent infections would result in hundreds of thousands of dollars in additional cost savings to insurers [17].

Use of a pneumatic compression device (PCD) was associated with decreases in the proportion of patients requiring hospitalizations, outpatient hospital visits, cellulitis treatments and physical therapy use [18].

Maintaining lymphedema in its early stages is associated with minimizing or even eliminating cellulitis [19]. It was observed that the incidence of infections decreased from 1.10 infections per patient per year to 0.65 infections per patient per year after a complete course of combined decongestive physiotherapy (CDP) [20].

Use of an advanced pneumatic compression device (APCD) improves health and reduces cost of treatment of lymphedema patients [21]. Notably, use of a PCD was associated with reductions in rates of cellulitis episodes in the cancer as well as in the noncancer lymphedema cohorts.

A recent cross-sectional study [22] included patients from 40 sites in nine countries, during the period of 2014–2017. Controlled swelling was associated with a 41% reduced risk of cellulitis. Cellulitis risk increased with the stage of lymphedema.

Lacking any credible population-based cost data, reticence of insurers to cover lymphedema treatment may be due to their belief that the costs of continued treatment of this chronic condition are large and uncontrollable. State legislators are reluctant to introduce lymphedema treatment mandates because of assurances by insurers that lymphedema is already being treated without a mandate. In responses to a Virginia Department of Insurance survey of Virginia insurers in 2003, 72% of the responders claimed to be treating lymphedema without the necessity of a mandate [23]. Similar survey responses were received from California healthcare insurers in 2005 [24] and Maryland insurers in 2017 [25]. But follow-up questionnaires in all three instances revealed that the treatment offered often fell short of the standards established by lymphedema specialty organizations such as American Lymphedema Framework Project, National Lymphedema Network, and the International Society of Lymphology [11].

In 2022 lymphedema treatment mandates exist in Virginia, North Carolina, and Maryland. A lymphedema insurance option is offered in Louisiana. And lymphedema treatment is mandated by the health codes of California and Vermont. Lymphedema treatment mandate bills have been submitted over the last decade in New York State and Massachusetts but have been stalled in legislative committees.

The Commonwealth of Virginia (COVA, Virginia, or VA) was the first state to introduce a lymphedema treatment mandate covering the cost of the treatment of lymphedema from all causes (not limited to breast cancer related lymphedema). The Virginia lymphedema mandate [26] became effective on January 1, 2004. It requires insurers, health services plans, and HMOs to provide “benefits for equipment, supplies, complex decongestive therapy, and outpatient self-management training and education for the treatment of lymphedema”.

This study of Virginia insurance claims data shows that insurance coverage costs of lymphedema treatment are reasonable, and that lymphedema treatment according to current medical standards results in health insurance contract savings due to reduced medical visits and hospitalizations. And if insurers continue to restrict lymphedema treatment coverage then State mandates may be necessary to help them save money.

## Methods

### Data sources

The Code of Virginia § 38.2–3419.1 requires every insurer, health services plan and health maintenance organization (HMO) that underwrites more than $500,000 of accident and sickness insurance subject to a State health mandate, to segregate and report to the State Corporation Commissioner the yearly (biennial since 2016) cost and utilization information for each of the 30 health mandates currently in effect. The Commission is required to prepare a consolidation of these reports for annual submission to the Governor and the General Assembly. This collection of annual reports [27], which includes the segregated annual costs of lymphedema treatment in Virginia, constitutes the most complete, non-proprietary population-based data set known to the author that documents the actual insurance cost of lymphedema treatment (Table 1).

**Table 1:** Data sources used in this study

Private, Group, HMO Contracts^a^			Pre-mandate Contracts^a^		
**Yr. Data**	**Yr. Rep’t**	**Rep’t No.**	**Yr. Data**	**Yr. Rep’t**	**Rep’t No.**
**2004**	**2005**	**RD191**	**1999**	**2001**	**HD007**
**2005**	**2006**	**RD289**	**2000**	**2002**	**HD010**
**2006**	**2007**	**RD246**	**2001**	**2003**	**HD008**
**2007**	**2008**	**RD322**	**2002**	**2003**	**RD049**
**2008**	**2009**	**RD294**	**2003**	**2004**	**RD110**
**2009**	**2010**	**RD300**	**State Employee Contracts**^b^		
**2010**	**2011**	**RD281**	**2009–10**	**2011**	**RD146**
**2011**	**2012**	**RD290**	**2010–11**	**2011**	**RD381**
**2012**	**2013**	**RD300**	**2011–12**	**2012**	**RD379**
**2013**	**2014**	**RD335**	**2012–13**	**2013**	**RD415**
**2014**	**2015**	**RD337**	**2013–14**	**2014**	**RD410**
**2015**	**2016**	**RD417**	**2014–15**	**2015**	**RD424**
**2016**	**2018**	**RD408**	**2015–16**	**2016**	**RD521**
**2017**			**2016–17**	**2017**	**RD588**
**2018**	**2020**	**RD471**	**2017–18**	**2018**	**RD510**
**2019**			**2018–19**	**2019**	**RD716**
**2020**			**1019–20**	**2020**	**RD655**

Reports available at Virginia’s Legislative Information system website https://rga.lis.virginia.gov/search/

^a^The Financial Impact of Mandated Health Insurance Benefits and Providers Pursuant To Section 38.2–3419.1 of the Code of Virginia: 20xx Reporting Period

^b^SFY20xx Mandated Benefits Report

In addition to the 14 annual and biennial mandate reports covering private insurance, group insurance, and HMOs, eleven separate annual reports were issued which document claim experience for State employee contracts for reporting periods starting with Fiscal Year 2010 (July 1, 2009 through June 30, 2010) representing an additional 5–6% of the Virginia insurance market (Table 1).

No attempt was made to combine the individual and State employee data as they contain somewhat different data and were collected over staggered time periods, i.e. private and group insurance and HMO data is for the calendar year while State insurance data is for the fiscal year.

#### Change in basis of reporting

The Virginia State Corporation Commission’s Rules Governing the Reporting of Cost and Utilization Data Relating to Mandated Benefits and Mandated Providers (the Rules) at 14VAC5–190-10 et seq., which specify the detail and form of the information that must be reported by companies, were changed, affecting data starting with 2016.

- The reporting period was changed to every other year, with each year in the period reported separately. However, the data from each reporting year was aggregated into one combined reporting period (i.e. 2016/2017, 2018/2019) and not displayed separately.
- The basic reporting unit was changed from “annual written premiums” to “covered lives”.
- HMOs and health services plans are not subject to all of the mandated benefit requirements of Title 38.2 of the Code of Virginia; however, the data provided by HMOs and health services plans starting with 2016 has been included in the data provided by insurers for the purposes of reporting claims costs and utilization as well as premium impact summaries.
- Units of coverage and percent of the market covered ceased being reported in 2014.

### Data collection

The data collection and reporting rules [28, 29] require insurers to use standard medical procedure and diagnosis codes when developing claim information for each benefit category. The codes utilized in the preparation of these reports are part of two widely accepted coding systems used by most hospitals, health care providers, and companies. These code systems are outlined in the Physicians’ Current Procedural Terminology (CPT-Plus) for medical procedures starting 2004, and the International Classification of Diseases - Clinical Modification 9th Revision (ICD-9-CM) for 2004–2014, and 10th Revision (ICD-10-CM) from 2015 onward for medical diagnoses (Table 2).

**Table 2:** Diagnostic and procedural codes collected

**Diagnostic Codes**	**2004-2015**^a^ **ICD-09-CM**	**2016–2019** **ICD-10-CM**
**Postmastectomy Lymphedema Syndrome**	**457.0**	**I97.2**
**Other Lymphedema**	**457.1**	**I89.0**
**Hereditary Edema of Legs/Hereditary Lymphedema**	**757.0**	**Q82.0**
**Procedural Codes**	**2004–2019** **CPT**	
**Massage, Compression**	**97124**	
**Manual Therapy Techniques, Manipulation**	**97140**	
**Self-Care/Home Management Training**	**97535**	

^a^Effective 30 Sep 2015 ICD-09 Codes Replaced with ICD-10 Codes

2015 reported data represents only 9 months

#### Claims

Claims are reported as dollar costs per contract or certificate. This instruction was clear until 2015. In 2016 a change in the reporting rules changed the basis for reporting from “annual written premiums” to “covered lives”. However, the description of the financial impact of the claims experience continued through 2019 to refer to the “average claim cost per contract or certificate” instead of the “claim cost per covered life”.

Instructions to companies filling out the input forms [28, 29] are explicit that the reported total claims used to determine percentage of total claims includes “all claims paid or incurred under the types of policies subject to the reporting requirements … and not the total claims paid or incurred for the mandate.”

#### Utilization of service

Insurers are required to report the number of medical office visits and the number of days of hospitalizations attributable to each mandated benefit for which claims were paid (or incurred) during the reporting period. This analysis focuses exclusively on group business because the group data is believed by the State to be significantly more reliable than that reported for individual business [27]. Utilization of services is represented in terms of the average number of visits per certificate for each mandated benefit, and the average number of inpatient or partial hospitalization days per certificate for each benefit.

#### Premium impact

Companies are required to use “actual claim experience and other relevant actuarial information” to determine the premium impact of each mandated benefit [27]. Because companies do not ordinarily develop separate rates for most benefits, much of the premium data reported to the Commission has been developed for the express purpose of complying with Virginia Code § 38.2–3419.1 [27]. The percent of overall average premium attributable to each mandated benefit is computed by dividing the estimated premium applicable to each mandated benefit by the overall average premium for all contracts subject to the reporting requirement.

Estimated premium impact is applied to an individual or family “Standard Policy” for a 30-year old male in the Richmond, VA area with a policy in the standard premium class including $250 deductible, $1000 stop-loss limit, 80% co-insurance factor and $250,000 policy maximum.

### Data analysis

Microsoft® Excel® for Mac Version 16.16.27 installed on the author’s Apple iMac under OSX Version 10.15.7 operating system was utilized to process the data and prepare the charts. The Excel built-in functions were utilized to determine the means, standard deviations, slopes, and ranges of the private and state population, claims, premiums, and utilization data.

## Results

### Population coverage

Over the 16 years considered in this study, 2004–2019, an average [range] of 19.3 [8-28] insurers and 11.4 [8-16] HMOs provided insurance coverage to 2.0–2.5 million Virginians each year. The portion of the insured population in Virginia covered by these reports (Table 1) approached 80%. Addition of reports for 2010–2020 for State-insured employees brings the coverage data to over 85% of the health insurance policies underwritten in Virginia.

### Claim costs for private insurers

**Fig. 1:**
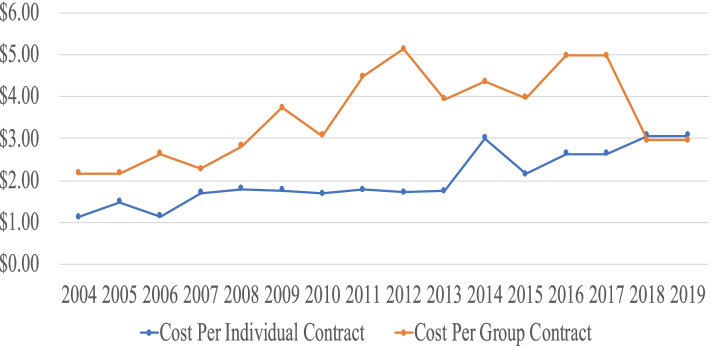
Lymphedema Claim Cost per Contract. Individual and Group Contracts

For the 16-year period of 2004–2019 the average annual lymphedema claim cost [and range] per individual contract was $2.03 [$1.12–$3.07], and per group contract $3.54 [$2.16–$5.13] (Fig. 1 and Table 3).

**Table 3:** 16-Year lymphedema claim statistics

**Cost Per Contract**	**Mean (USD)**	**Standard Deviation (USD)**	**Slope (% per Year)**
**Individual Contract**	**$2.03**	**$0.63**	**+6.05**
**Group Contract**	**$3.54**	**$1.00**	**+3.59**
**Percent of Total Claims**	**Mean (%)**	**Standard Deviation (%)**	**Slope (% per Year)**
**Individual Policies**	**0.05**	**0.008**	**-1.26**
**Group Policies**	**0.08**	**0.018**	**-1.52**
**HMO Individual Policies**^a^	**0.01**	**0.011**	**+13.57**
**HMO Group Policies**^a^	**0.04**	**0.017**	**+7.93**

^a^12 Year’s Data

Average claim costs for lymphedema treatment exhibited a 3–6% annual growth over the 16 years of the Virginia mandate for both individual and group policies (Table 3). The growth trends, however, displayed different characteristics (Fig. 1**).** Individual contract lymphedema claims displayed an initial growth the first 3 years, after which they remained virtually constant through 2013 at around $1.75 per contract per year, then rose to $3.07 by 2019, averaging 6.05% increase per year over the report period. Group contract lymphedema claims, while higher than for individual contracts, displayed an uneven growth over the sixteen-year period averaging a lower 3.59% per year.

### Claim costs for state employee contracts

**Fig. 2:**
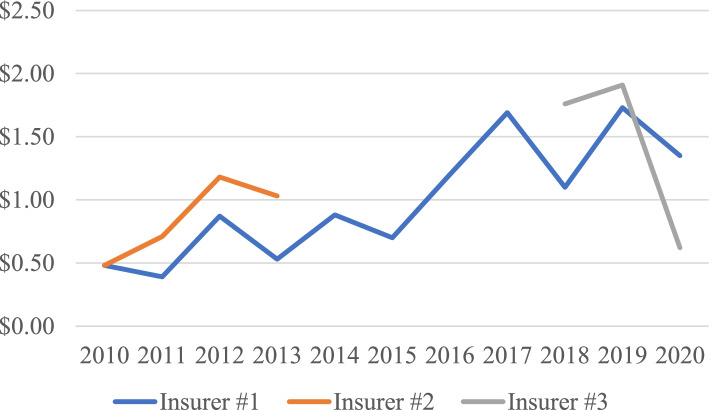
Lymphedema Claim Costs per Contract. State Employee Contracts

Annual data was collected from three insurers through Fiscal Year 2020 (Fig. 2). For Insurer #1 average lymphedema costs per contract were $0.90 [$0.39–$1.73] for an average of 81,664 individual insureds per year over the period 2010–2020. Data for Year 2016 was excluded since it was invalid due to transition in diagnostic codes, and collection did not include a full 12 months. Claim costs per contract for Insurer #2 for years 2010–2013 was $0.85 [$0.48–$1.18] for 8119 group members. and for Insurer #3 for years 2018–2020 was $1.43 [$0.62–$1.91] for an average of 15,828 contracts. Data reported for years 2014–2017 was reported using different methods and populations, and cannot be included in any trend analysis. State employee contract claims rose from around $0.50 in 2010 to $1.50 in 2020 (Fig. 2), remaining, however, well below the claim costs for individual and group contracts (Fig. 1).

### Lymphedema claims as a percentage of Total contract claims

**Fig. 3:**
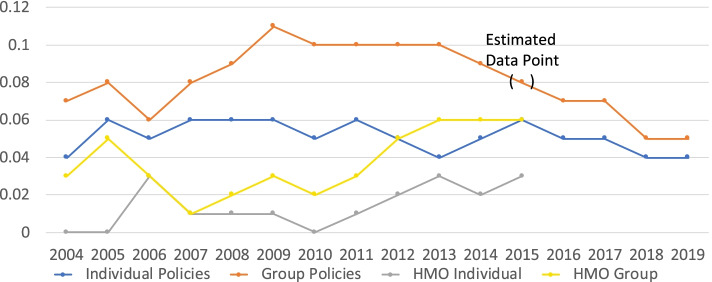
Lymphedema Claims as a Percentage of Total Contract Claims

The lymphedema claims as a percentage of the total contract claims filed for individual contracts was 0.05% [0.04%–.06%], and for group contracts was 0.08% [0.05–0.11%] (Fig. 3 and Table 3). The percentage of lymphedema claims to all HMO contract claims was lower, averaging 0.01 and 0.04% for individual and group contracts (Fig. 2 and Table 3). HMO data was not separately reported after 2015.

### Utilization

**Fig. 4:**
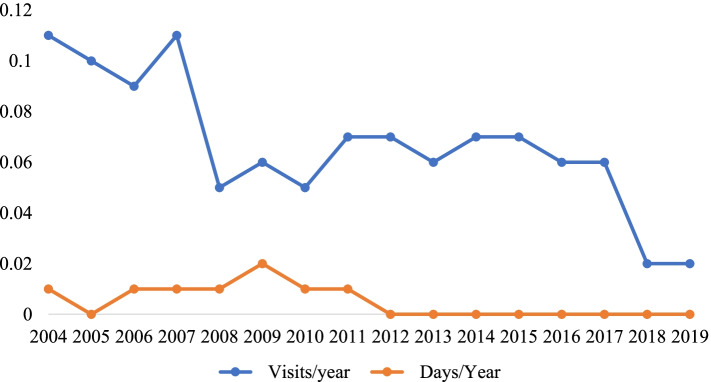
Medical Office Visits and Hospitalizations, Group Certificates

The number of visits for lymphedema treatment averaged 0.10 [0.09–0.11] visits per year for the first 4 years of the mandate (Fig. 4), dropped to 0.06 [0.05–0.07] visits per year for the next 10 years, and finally to 0.02 visits per year in the last 2 years. The mean number of provider visits for lymphedema treatment per year per contract for the three State insurers ranged from 0.0016 to 0.0052. Hospitalizations for lymphedema remained at or below 0.02 days per year per contract during the entire 16-year period (Fig. 4). Average hospitalizations for two of the State insurers ranged from 0.0002 to 0.0012 days per contract per year. Information for the third insurer was not reported.

### Lymphedema premium allocation

The annual premium allocated to lymphedema for individual and group contracts over the period of 2004–2019 fluctuated between 0.64 and 0%.

**Fig. 5:**
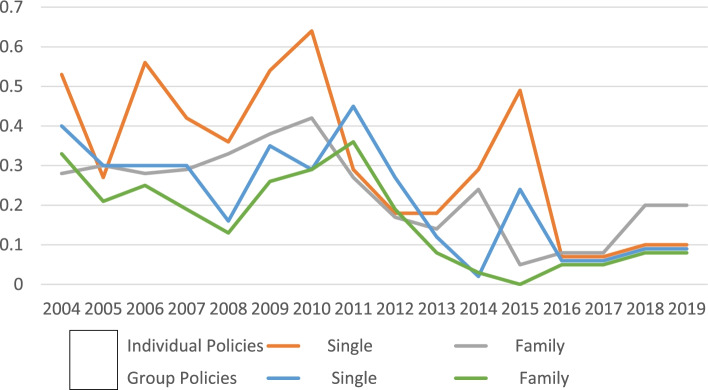
Lymphedema Premium Allocation Percent of Contract. Individual and Group Contracts

**Fig. 6:**
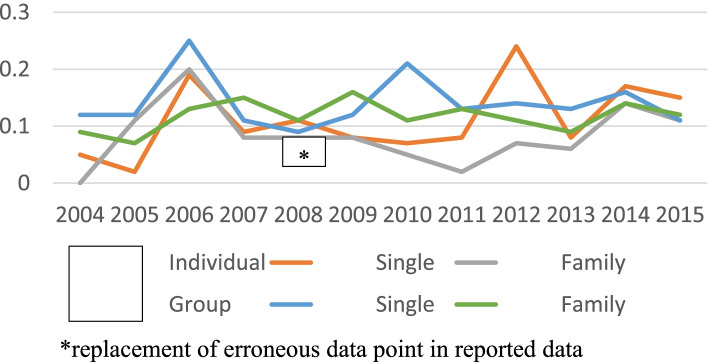
Lymphedema Premium Allocation Percent of Contract. HMO Contracts. *replacement of erroneous data point in reported data

The estimated premium impact of lymphedema treatment (Figs. 5 and 6) ranged 0.14 to 0.64% of the overall average contract premium on individual and group contracts, and 0.00 to 0.25% on HMO contracts. There was a general trend over the period for all types of contracts after 16 years of operational experience toward 0.1–0.2%. (Fig. 5).

The portion of annual HMO premiums attributed to lymphedema treatment was relatively stable over the 2004–2015 reporting period, trending toward 0.15% of total average premium. (Fig. 6).

## Discussion

From 2008 to 2012 the number of persons in Virginia under the age of 65 with private health insurance decreased 3.0% while public coverage (e.g. Medicaid) increased only 1.3% [30].

The number of persons covered fluctuated between 2.0 and 2.4 million, covered by approximately 1.1 million certificates each year. Independent data from insurance company filings with the National Association of Insurance Commissioners over the years 2010–2020 indicate that the ratio of “covered lives” to “contracts or certificates” was 1.8–2.0. This compares with Census data indicating 2.6 persons per family in Virginia 2016–2020. Changes in reporting procedures from “group contracts” to “covered lives”, and the inclusion of HMO data with individual coverage in 2016, accounts for an increase in coverage units from 1.1 million contracts or certificates to over 2 million covered persons.

The actual numbers of companies filing reports showed a steady decline from 28 to 16 over this period, following observed trends by other researchers. The number of reporting companies dropped from 24 to 28 to 17–20 after 2010, a possible indication of the healthcare industry consolidation starting after passage of the Patient Protection and Affordable Care Act of 2010 [31]. Another significant reduction in the number of insurers reporting in 2016 from 17 to 20 to 8 may be related to the reporting simplification changes starting in 2016, which allowed major insurers to combine reporting of private and HMO statistics and revised the definition of who must file biennial reports.

**Fig. 7:**
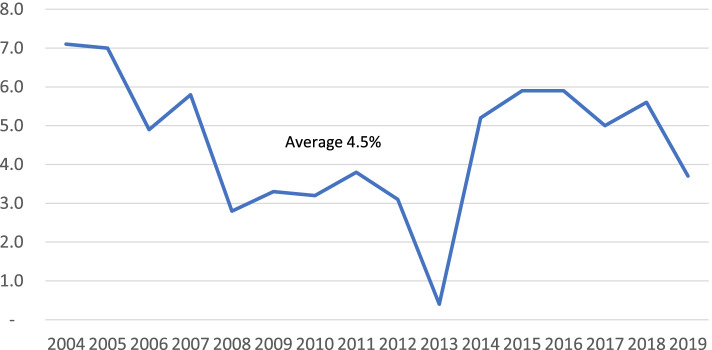
Annual Percent Change in National Health Expenditures. Private Health Insurance*. *Table 3https://www.cms.gov/Research-Statistics-Data-and-Systems/Statistics-Trends-and-Reports/NationalHealthExpendData/NationalHealthAccountsHistorical

Growth in claims costs of 3–6% per year generally match the 4.5% growth in annual national health expenditures over the 2004–2019 period (Fig. 7). Neither lymphedema office visits nor hospitalizations (Fig. 4) history support the idea that increased utilization was responsible for the rising claim costs. The reduction of group contract claim cost from $4.97 to $2.96 in the 2018/2019 report may be due to the revised definition of the basic insurance unit from “written premium” to “covered lives”. The “cost per group contract” thereby changed to “cost per covered life”, making that measure similar to the cost per individual contract of $3.07.

Lymphedema claims, over the 16 years of the study, represented less than 0.1% of the total contract claims (Fig. 3), and approached 0.04–0.05% in the last year reported. A series of annual mandate reports for the 5 years preceding the introduction of the lymphedema mandate was examined to determine whether introduction of lymphedema treatment affected healthcare cost to any significant degree. None was evident.

Virginia insurance companies are required to use “actual claim experience … and other relevant actuarial information” to determine the premium impact of each mandated benefit [27]. While mean lymphedema claims represent 0.05 to 0.08% of total claims (Table 3), the portion of the total contract premium attributed to lymphedema was four times higher, i.e. 0.16 to 0.32% of the average contract premium. This discrepancy led to a thought experiment to explain the premium spikes observed in Figs. 5 and 6, and the differences in the year-to-year premium allocation with respect to lymphedema treatment claim percentages.

**Fig. 8:**
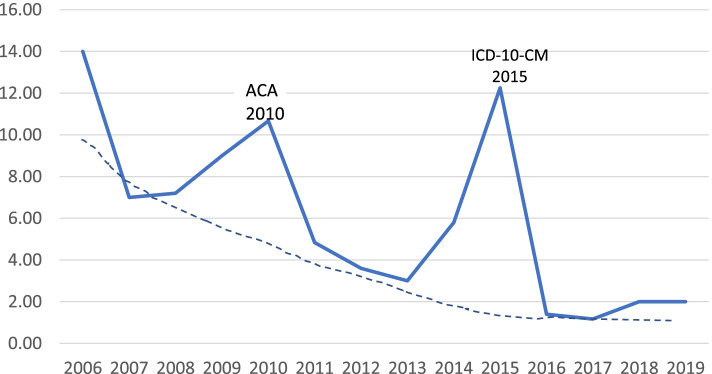
Ratio of Lymphedema % Premium (Year n) to % Claims (Year n-2)

Assuming that premiums for year “n” are determined by underwriters in year “n-1” based on the claims paid in year “n-2”, the ratio of premium allocation to lymphedema treatment in year “n” to claims paid year “n-2” (as percentages of total average premiums and total claims respectively) should be a rough measure of the underwriters’ estimated risk for the lymphedema mandate. For the case that claims as a percent of total claims is well known, and future risk of change in lymphedema claims is low, premium percent of total premium should be of the same order as claims percent of total claims—or the ratio of the premium-to-claims percentages should be unity. This ratio is plotted in Fig. 8. The three peaks represent the anticipated actuarial risks in anticipation of: 1. introduction of the new lymphedema mandate in 2004 with no claims history; 2. introduction of the PPACA in 2010; and 3. transition of diagnostic codes from ICD-9-CM to ICD-10-CM in 2015. In these 3 years premiums were elevated from a more normal learning curve for a new mandate--a learning curve which should approach 1.00 where premiums are allocated on the sole basis of claim history.

The reductions in lymphedema-related physician visits and hospitalizations over the 16-year lymphedema treatment period is a notable trend, verifying the basic tenet that treatment of lymphedema reduces the incidence of infections requiring medical attention and hospitalization for upper and lower extremities, in cancer- and non-cancer-related lymphedema.

The reduction of mean visits per year per contract in 2018 from 0.06 to 0.02 may be due to the revised definition of the basic insurance unit from “written premium” to “covered lives” (i.e. “medical office visits per group certificate” changed to “medical office visits per covered life”). According to the U.S. Census Bureau the average number of persons per household in Virginia 2016–2020 was 2.6.

It is not known why utilization rates for the State employee contracts are an order of magnitude lower than for individual or HMO contracts. Coverage requirements, established by statute, apply equally to private and State contracts.

### Errors and limitations

#### Changes in reported data

On February 13, 2017, by order of the Virginia State Corporation Commission, certain changes were made to the rules for reporting cost and utilization data relating to mandated benefits. These changes have the potential of introducing singularities into, and affecting trend analysis of, collected data. Reporting frequency was changed from annual to biennial. Costs were reported by insurers to the State of Virginia using Form MB-1 [27] through 2015, and electronic Form 190A [28] thereafter. Information on units of coverage and percentage of the Virginia insurance market were no longer reported. HMO data was combined by providers with other of their health insurance and health services.

The change in reporting rules from contract to covered lives was supposed to have become effective in the 2016/2017 report, but inspection of the data which is normalized to contract/certificate or covered lives suggests that the actual change was not applied until the 2018/2019 report. Consider the following three examples:

- Lymphedema claim cost per contract for individual contracts in 2018/2019 (Fig. 1) fell to about half of the value it would have had if it had continued its previous rising trend from 2004;
- Lymphedema claim costs per contract in 2020 for Insurer #3 (Fig. 2) fell to a fraction of its previous values from 2018/2019;
- Medical office visits per certificate in 2018/2019 (Fig. 4) fell to one third of its value from 2008 to 2017.

#### Completeness of reported mandate costs

The covered benefits for lymphedema treatment include “equipment, supplies, complex decongestive therapy, and outpatient self-management training and education” [26].

The Virginia Insurance Bureau delineates the specific diagnostic codes to be collected in the reporting of lymphedema claim costs [29]. The codes collected and priced in the company claims reports must include ICD-9-CM/ICD-10-CM lymphedema diagnostic codes: 457.0 / I97.2 Postmastectomy lymphedema syndrome; 457.1 / I89.0 Other lymphedema; and 757.0 / Q82.0 Hereditary edema of legs/hereditary lymphedema. In their instructions to insurers, the Bureau of Insurance notes that “the CPT and ICD-9-CM codes are not intended to exhaust all medical codes that may be used in collecting data for Form MB-1, but are representative of some of the more common codes associated with the mandated benefits”. While these codes are the only diagnostic codes specifically mentioned in the instructions, it is not clear whether other lymphedema codes such as: 624.8 / N90.89; 374.83 / H02.851–9; or 997.99 / I97.89 for lymphedema of body sites other than the arm or leg, or caused by surgery, are included.

The instructions are not explicit as to what costs are to be included except to request “specific claim data” for each mandated benefit. Specific CPT codes are required to be collected include the physical therapy CPTs 97124, 97140 and 97535 [29]. This covers massage therapy, manual therapy, and outpatient self-management training and education. Lymphedema treatment also includes: costs associated with physician and therapist evaluation; costs of compression bandages, garments, devices and supplies used in a home setting in the daily management of lymphedema; vasopneumatic device therapy; and therapeutic exercises, when used for the treatment of lymphedema or for lymphedema patient instruction. It is not clear whether charges for these procedures are uniformly collected and reported.

#### Data errors in diagnostic code transition year

Two data errors became apparent upon inspection of the tabular data from the annual reports. These data points fell outside of 3-sigma values for the measurements over the reported periods, and both occurred in the reports for 2015–2016 claims (i.e. 2015 group claim percentage of total claims, and 2016 state health benefit claim cost per contract) and were probably related to the diagnostic code transition data collection. A warning was noted in the Executive Summary of the 2015 data report 2016-RD417 about the “uncertainty underlying combinability of the data”. A similar notice appeared in the cover letter for the State employee insurance report 2016-RD521. The relevant data points were adjusted by the author to be halfway between adjacent year values (Figs. 3 and 2).

#### Data error in 2009 annual Report

An obvious error was found in the Premium Impact Summary for HMO Individual Family contracts in Report 2009-RD294. The incorrect value of 3.00% was replaced by the author by adjacent year value of 0.08% (Fig. 6).

## Conclusions

An estimate of the cost of lymphedema treatment from an insurer’s viewpoint was made using 16 years of actual claims experience in Virginia, where a lymphedema treatment mandate has been in effect since January 1, 2004.

Compilation and analysis of the Virginia lymphedema mandate data was made 5, 7, and 10 years since the start of the mandate [32–34]. The 10-year report [34] included comparisons of the Virginia mandate with mandates proposed and passed for other states. This current analysis adds 6 years of experience and covers the transition of the diagnosis code from ICD-9-CM to ICD-10-CM and additional experience under the Patient Protection Affordable Care Act (ACA).

Claim experience is a direct measure of the cost of lymphedema treatment, and is the focus of this study. The salient conclusion is that lymphedema treatment costs are less than one thousandth of the total claims costs in all insurance contract types. In so far as most Virginia insurers claim to have been treating lymphedema before the mandate, and the reports gather all lymphedema treatment costs by medical diagnostic code, the reported claims represent the total cost for lymphedema treatment rather than the incremental increase due to the mandate.

After 16 years of actuarial experience with the lymphedema mandate the premium impact for all types of contracts converged to less than 0.2% of the average contract total premium. The estimated premium attributable to lymphedema treatment are many times the actual claims experience for the years analyzed, probably reflecting an actuarial increase in premium in anticipation of a perceived risk.

The Virginia lymphedema treatment mandate and associated cost-benefit reporting provide an unprecedented public source of authoritative data which demonstrates that lymphedema treatment coverage costs are reasonable, and result in significant savings in medical care and hospitalizations by prevention of lymphedema-related cellulitis and progressive disability. The Virginia data confirmed previous study conclusions that the treatment of lymphedema by management of swelling results in lower medical costs and fewer hospitalizations. This is a potent model for reduction in healthcare costs while improving the quality of care for cancer survivors and others suffering with this chronic progressive condition.

## Data Availability

The datasets used and/or analyzed during the current study are available from the author on reasonable request. The dataset comprises abstracts from publicly-available annual reports enumerated in Table 1.

## Abbreviations

ACA: The Patient Protection and Affordable Care Act (Also PPACA)
ALFP: American Lymphedema Framework Project
APCD: Advanced pneumatic compression device (HCPCS code E0652)
BCRL: Breast cancer-related lymphedema
CDT: Complex or combined decongestive therapy (Also CDP or CPT)
CM: Clinical Modification (Used with ICD, i.e. ICD-10-CM or ICD-9-CM)
CI: Confidence interval
COVA: Commonwealth of Virginia (Also State of Virginia, Virginia, or VA)
CPT: Physicians’ Current Procedural Terminology
CPT: Combined physiotherapy (Also CDT complex decongestive therapy)
DLA: Dermatolymphangioadenitis or cellulitis
FY: Fiscal year (i.e. July 1 – June 30)
HCPCS: Healthcare Common Procedure Coding System
HMO: Health maintenance organization
ICD: International Classification of Diseases (e.g. ICD-10-CM or ICD-9-CM)
ISL: International Society of Lymphology
NLN: National Lymphedema Network
OR: Odds ratio
PCD: Pneumatic compression device (HCPCS code E0651 or E0652)
PPACA: The Patient Protection and Affordable Care Act (Also ACA)
VA: Virginia, or Commonwealth of Virginia, or State of Virginia
